# A simple personalised prediction model for hip fracture risk based on mechanistic parameters

**DOI:** 10.1007/s10237-026-02051-z

**Published:** 2026-03-30

**Authors:** Christina Wapp, Yvan Gugler, Paula Cameron, Alice Dudle, Daniela Frauchiger, Maria Papageorgiou, Emmanuel Biver, Serge Ferrari, Kurt Lippuner, Philippe Zysset

**Affiliations:** 1 ARTORG Center for Biomedical Engineering Research, University of Bern, Bern, Switzerland; 2 Division of Bone Diseases, Department of Medicine, Geneva University Hospitals and Faculty of Medicine, University of Geneva, Geneva, Switzerland

**Keywords:** Hip fracture, Fracture risk, Fragility fracture, Falls, Impact force, Prediction

## Abstract

Fragility hip fractures in older adults are a major burden for the individual and the society, as they result in increased morbidity, mortality and substantial health care expenditure. Most fragility hip fractures are caused by falls, resulting in an impact on the postero-lateral aspect of the hip, inducing a force that exceeds the femoral bone strength. The risk of fracture depends on the rate of falling, the fall-induced impact force, and the femoral bone strength. This article presents the framework of a novel fragility hip fracture risk calculator based on a combined mechanistic and stochastic modelling approach. The model integrates the stochastic aspects of a fall and its dynamics together with a 1D mechanical model predicting the impact force in the hip to calculate a one-year absolute fracture risk. The required input parameters are based on QCT images, but can be substituted with more accessible anthropometric and densitometric data. To demonstrate how the parameters are extracted from clinical data and how the model is calibrated, data from the AFFIRM-CT cohort were used. A sensitivity analysis was conducted, confirming that the fall rate, the trochanteric soft tissue thickness and the bone strength are the dominating parameters influencing the risk of fragility hip fractures. Furthermore, output variables such as the predicted impact velocity and impact force aligned well with experimental data. Thus, the model is able to reflect observations from empirical data, indicating that it can capture the intrinsic aspects that define the risk of fragility hip fractures.

**Supplementary Information:**

The online version contains supplementary material available at https://doi.org/10.1007/s10237-026-02051-z.

## Introduction

In 2019, around 82’000 fragility fractures were registered in Switzerland in individuals aged 50 and older, referring to an annual fracture rate of 23.5 fractures per 1000 individuals (Kanis et al. 2021). Due to the ageing of the population, this number is expected to increase by 37.5% by 2034. The consequences of fragility fractures include morbidity, mortality, and substantial health care expenditure (Hernlund et al. 2013; Svedbom et al. 2014; Veronese and Maggi 2018). Fractures of the hip are the most serious type, with a mortality risk of up to 20% (Marks et al. 2003). In this context, it is of great interest to identify individuals at risk of fragility fractures, especially of the hip.

Fragility fractures are the primary clinical outcome of osteoporosis, a silent metabolic bone disease affecting primarily older adults. Osteoporosis is characterised by bone loss (Compston et al. 2019) and can be assessed by measuring areal bone mineral density (aBMD) with dual energy X-ray absorptiometry (DXA) (Dimai 2017). FRAX®  is a frequently used model for fracture risk prediction that combines clinical risk factors with or without aBMD using a statistical regression model (Kanis et al. 2007, 2008), as aBMD alone has limited predictive power at the individual patient level (Marshall et al. 1996; Kanis et al. 2002; Hernlund et al. 2013).

When looking at the mechanisms leading to fragility hip fractures, it is found that around 95 % of the cases are caused by falls resulting in an impact on the postero-lateral to lateral aspect of the hip (Parkkari et al. 1999; Court-Brown et al. 2017; Yang et al. 2020). From a biomechanical point of view, fractures occur when the impact force exceeds bone strength. Since only 1–3 % of all falls result in a fracture (Yang et al. 2020; Komisar and Robinovitch 2021), this suggests that either the available energy is often not sufficient to cause a fracture and/or that energy-dissipating mechanisms reduce the impact force below a critical level (Komisar and Robinovitch 2021). Thus, the risk of hip fracture is dependent on different factors that can be summarised by three main points, namely (1) the actual risk of falling, respectively the frequency of falls, (2) the impact force on the hip and (3) the femoral bone strength.

aBMD values from DXA can be used to estimate bone strength, as these two measures are known to strongly correlate (Bouxsein et al. 2020). Alternatively, bone strength can be computed with finite element analysis (FEA) based on quantitative computed tomography (QCT) images, a method that has been thoroughly studied and validated (Engelke et al. 2016; Bouxsein et al. 2020). The assessment of bone mineral density (BMD) and fracture risk with the use of clinical QCT scans that were performed for other diagnostic purposes is known as opportunistic screening (Engelke et al. 2023).

The magnitude of the impact force directly depends on the impact velocity and the visco-elastic behaviour of the hip complex. The impact velocity in turn relies on different factors such as the initial height of the fall and the effective mass, the pre-impact movement strategies or the landing configuration (Yang et al. 2016, 2020; Komisar and Robinovitch 2021). These aspects of fall dynamics can vary significantly between fall events and are difficult to predict. The visco-elastic behaviour of the hip determines the force attenuation during the transmission through the body, and thus determines the force magnitude reaching the femur. Various *in vivo* and *ex vivo* experiments were conducted to characterise the behaviour of the hip complex upon impact (Robinovitch et al. 1991; Laing and Robinovitch 2010; Levine et al. 2013; Fleps et al. 2019), and damped vibrational systems were used to model and predict the impact response (Robinovitch et al. 1997). Thereby, the trochanteric soft tissue, respectively its thickness, was shown to be a critical factor influencing the effective stiffness and damping properties (Robinovitch et al. 1991, 1995; Bouxsein et al. 2007; Majumder et al. 2008). Other parameters that influence these properties are the condition of the muscles (relaxed/contracted) (Robinovitch et al. 1991; Kim et al. 2023) or the floor type (Laing et al. 2006).

Various mechanistic models have been developed to estimate the peak impact force integrating aspects of the fall dynamics and/or the visco-elastic behaviour of the hip (Sarvi and Luo 2017; Luo et al. 2014; Sarvi and Luo 2015; Fleps et al. 2019; Palanca et al. 2021; Dufour et al. 2012). The predicted impact force can then be compared with bone strength, which is known as the load-to-strength ratio or the factor of risk (Dufour et al. 2012; Sarvi and Luo 2017). However, this ratio does not inform about a fracture risk, but allows for the distinction between fracture and non-fracture cases. To our knowledge, only few mechanistic models that predict an absolute fracture risk have been presented in the literature. For instance, Bhattacharya et al. developed a multi-scale model that integrated a mechanistic model into a probabilistic setting, calculating a one-year fracture risk (Bhattacharya et al. 2019).

In 2010, an alternative modelling approach for hip fracture risk, distinct from those previously reported in the literature, was presented in a publication that gained surprisingly little attention in the research community (Schechner et al. 2010). The model is based on a mechanistic stochastic framework and derives the probability of at least one hip fracture in a given time interval (0,T] by combining the stochastic aspects of a fall and the fall dynamics with femoral bone strength. Briefly, the occurrence of a fall is modelled with a Poisson process characterised by the rate parameter λ. The model then filters the fall events resulting in a fracture by introducing a conditional probability, modelled as a random variable, that describes the chances of the fall-induced impact force exceeding the femoral bone strength. Without the use of any clinical data, the model is able to describe key aspects of hip fractures observed in clinics, such as the limited predictive power of BMD for the individual due to the strong overlap between the BMD distributions of fracture and non-fracture individuals, or that a prior hip fracture results in an increased fracture risk. Given these findings, the model appears to offer a promising foundation for a mechanistically motivated hip fracture risk calculator. However, the article only presents the mathematical framework for a generic population and lacks patient-specific input variables and personalisation.

The goal of our work was to further develop and personalise the model suggested by Schechner et al. by incorporating patient-specific input variables, making it applicable at the individual level. In previous work, we have developed a model to predict a personalised fall rate λ (Wapp et al. 2022, 2023, 2024). Here, with the aim of more accurately characterising the conditional probability of a fracture given a fall, we present a model that predicts a personalised impact force using subject-specific parameters, which is then combined with a probability distribution that describes the stochastic nature of the fall dynamics. The required parameters are mostly derived from QCT images, with substitution equations provided for cases where such images are not available. As a major outcome, a sensitivity analysis to assess the influence of the different parameters of the calculator on the fracture risk was conducted. To demonstrate how the required model parameters can be extracted from clinical data and how the model is calibrated, data from the AFFIRM-CT cohort were used as an example.

## Methods

### Impact force model

The probability of at least one hip fracture in the time interval (0, *T*] is given by a thinned Poisson process such that

1 $$\begin{aligned} P({\textit{Fracture in} } (0,T]) = 1-e^{-\lambda p_s T} \end{aligned}$$

with λ as the fall rate, and $p_S=P(Fracture|{\textit{Fall in} } (0,T])$ as the conditional probability of a hip fracture given a fall (Schechner et al. 2010). A detailed description of the derivation of this formulation can be found in the supplementary material or in the original publication by Schechner et al. (2010). The conditional probability of a fracture given a fall $p_S$ is defined as the probability of the fall-induced load exceeding femoral strength, with the load modelled as a random variable accounting for the varying fall dynamics. In the original publication, a broad assumption was made about the probability distribution of this random variable. In the following sections, we present a personalised impact force model characterising $p_S$. We introduce a mechanical 1D mass-spring-damper system consisting of rheological elements representing the hip complex and the ground. It models the non-linear visco-elastic response of the hip upon impact, allowing the calculation of the impact force in the hip. Thereby, the impact velocity is the critical factor that defines the magnitude of the impact force. Since the impact velocity varies with the fall dynamics, a second model that derives the range of possible impact velocities given different fall scenarios is introduced. And last, we present a probability distribution that describes the probability of different impact velocities given the fall dynamics, allowing for the calculation of an absolute fracture risk Table 1.

**Table 1:** List of symbols and abbreviations for the hip fracture risk calculator in alphabetic order

Symbol/abbreviation	Meaning
*cmh*	Height of the centre of mass at the start of the fall measured from x=0
*cmi*	Height of the centre of mass at the start of the impact measured from x=0
$F_{()}$	Forces in the rheological elements of the ground (G), soft tissue (ST), femur and hemi-pelvis (HP)
$fas_{S0}$, $fas_{S1}$	Spring force amplitude of the soft tissue
*FSR*	Ratio between the fall-induced peak force applied to the femur $F_{Femur}$ and the femoral strength $F_{Strength}$
$h_{()}$	Initial height of the ground (G), soft tissue (ST), femur and hemi-pelvis (HP)
*height*	Full body height
$k_{()}$	Stiffness of the rheological elements of the ground (G), soft tissue (ST), femur and hemi-pelvis (HP)
$k_{BR_{crit}}$	Critical stiffness of the body resistance, resulting in a FSR=1
*m*	Effective mass
$p_S$	Conditional probability of a fracture given a fall
$rk_{BR}$	Relative value of the stiffness of the body resistance so that $rk_{BR} \in [0,1]$
*T*	Time interval of the Poisson process
$t_{imp}$	Time at impact with t=0 at the start of the descent phase of a fall
$v_{imp}$	Velocity at impact, when the hip touches the ground
*weight*	Full body weight
α	Exponent of the probability distribution describing the fall dynamics
λ	Fall rate characterising the Poisson process
$\mu _{D1}$	Soft tissue damping coefficient

#### Mechanical model

**Fig. 1:**
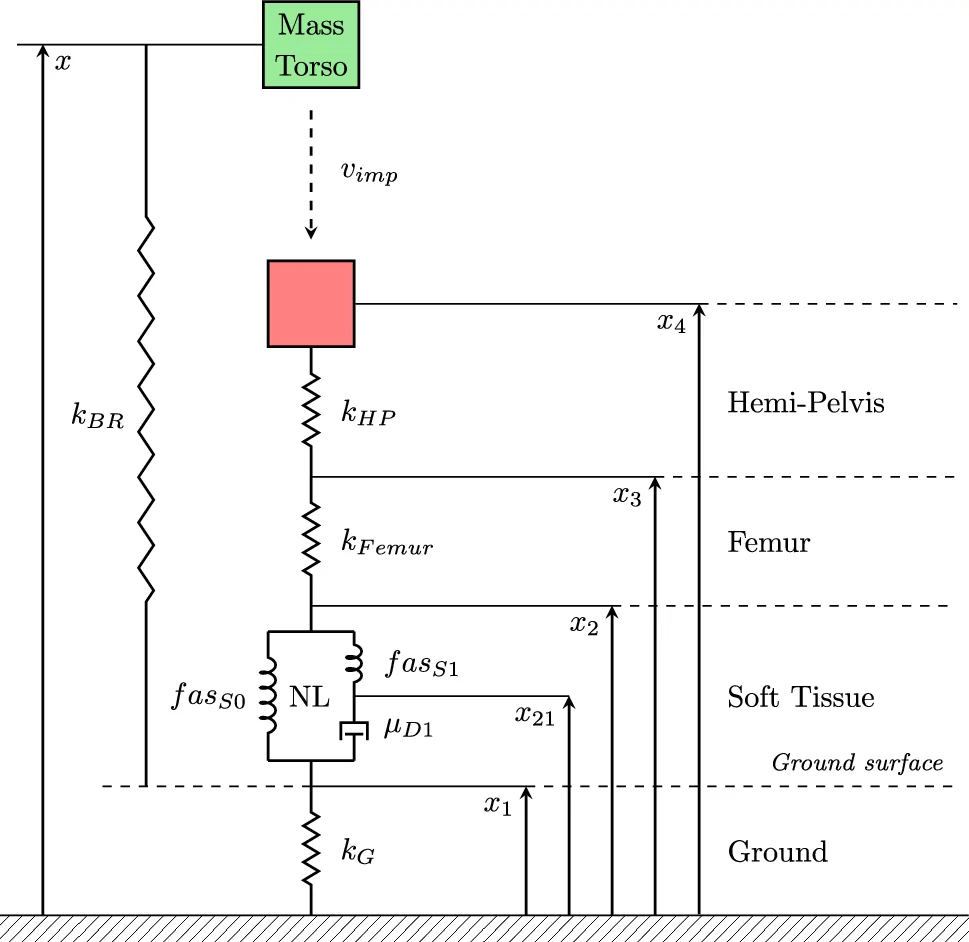
Drawing of the impact force model: The fall dynamic model consists of the spring $k_{BR}$, which is used to calculate the impact velocity. The mechanical 1D model consists of rheological elements representing the hip complex and the ground. It requires the impact velocity as defined by the fall dynamics model as an initial condition

The mechanical model is a 1D model that reflects a standardised postero-lateral impact on the hip. It is composed of rheological elements representing the pelvic ring, the femur, the soft tissue and the ground. As the centre of mass at the impact is assumed to be in the centre of the torso, the model only depicts half of the hip. The hemi-pelvis (HP), the femur and the ground (G) are modelled with linear springs, and the visco-elastic behaviour of the soft tissue (ST) is represented by a standard non-linear solid (Fig. 1).

The equations of motion of the mechanical model are given as a system of differential algebraic equations (DAE) such that

2 $$\begin{aligned}&\nonumber m*x_4'' + F_{BR} + F_{HP} + m*g = 0\\&\nonumber F_{HP} - F_{Femur} = 0\\&\nonumber F_{S0} + F_{S1} - F_{Femur} = 0\\&\nonumber F_{S1} - F_{D1} = 0\\&F_{S0} + F_{S1} - F_{G} = 0 \end{aligned}$$

with $F_{()}$ as the rheologic elements’ corresponding forces that depend on the variables $x_1(t)$, $x_2(t)$, $x_{21}(t)$, $x_3(t)$, $x_4(t)$, and m=0.5∗0.678∗weight as the effective mass of the upper body (van den Kroonenberg et al. 1995) and $g = 9.81 m/s^2$ as the gravitational acceleration. The initial conditions of the DAE system are defined as follows:

3 $$\begin{aligned} \nonumber x_4'(0)&= -v_{imp}\\ \nonumber x_4(0)&= h_{G} + h_{ST} + h_{Femur} + h_{HP}\\ \nonumber x_3(0)&= h_{G} + h_{ST} + h_{Femur}\\ \nonumber x_{21}(0)&= h_{G} + h_{ST}\\ \nonumber x_2(0)&= h_{G} + h_{ST}\\ x_1(0)&= h_{G} \end{aligned}$$

with $v_{imp}$ as the impact velocity and $h_{()}$ as the initial height of the respective rheological elements. By solving the DAE for $x_1(t)$, $x_2(t)$, $x_{21}(t)$, $x_3(t)$ and $x_4(t)$, the impact force can be calculated. Thereby, the impact velocity $v_{imp}$ is the only initial condition that changes depending on the fall dynamics. Thus, it is the critical factor that determines the magnitude of the impact forces in the mechanical systems. The explicit formulations of the forces can be found in the supplementary materials.

#### Fall dynamics model

We decided to model the fall dynamics resulting in different impact velocities with a linear spring representing the resistance of the body (BR) to the fall (Fig. 1). Thereby, the spring, characterised by the constant $k_{BR}$, acts as an energy storage mechanism during the fall and the impact, with more energy stored resulting in a lower impact velocity. The spring constant represents all parameters that can vary from fall to fall and that are difficult to quantify. This includes different fall scenarios and pre-impact movement strategies, e.g. the initial height of a fall, a rotational movement during the descent, the activation of muscles, or the use of an outstretched hand to dampen the fall, which may, in particular, be represented by the parallel spring. But $k_{BR}$ also accounts for other parameters of the 1D mechanical model that vary from fall to fall and thus are not realistically reflected by a standardised postero-lateral fall, as, for example, the influence of impact site and orientation on the soft tissue thickness (Lafleur et al. 2021).

To cover all possible fall scenarios of an individual, $$k_{BR}$$ can be any number within its physically defined range. Thereby, the upper limit of the range was derived by equating an individual’s potential energy to the elastic potential energy of a spring, resulting in $$k_{BR_{max}} = \frac{-2*m*g}{cmi-cmh}$$ with $$cmh = 0.53*height + h_{G}$$ as the height of the centre of mass at the start of the fall when standing (van den Kroonenberg et al. 1995), and $$cmi = h_{HP} + h_{Femur} + h_{ST} + h_{G}$$ as the height of the centre of mass at the start of the impact. This represents a fall scenario in which all potential energy is stored in the spring, resulting in an impact velocity of $$v_{imp} = 0$$. The lower limit of the spring constant was set to $$k_{BR_{min}} = 0$$, representing a fall scenario in which all potential energy is converted into kinetic energy. Accordingly, the maximum impact velocity is reached when the spring constant is set at its minimum.

The impact velocity can be obtained from the differential equation $$m*x''+k_{BR}*(x-cmh) + m*g = 0$$ with the initial conditions x(0)=cmh and $x^{\prime }\left(0\right)=0$. It is given by

4 $$\begin{aligned} v_{imp} = -\sqrt{\frac{m}{k_{BR}}} * g*sin\bigg (\sqrt{\frac{k_{BR}}{m}}*t_{imp}\bigg ) \end{aligned}$$

with $t_{imp}$ as the time at impact, defined as the time of the hip touching the ground. The detailed derivation can be found in the supplementary material.

Consequently, the spring constant $k_{BR}$ is the critical parameter determining the impact velocity of the hip. The impact velocity, in turn, defines the magnitude of the impact force in the hip complex and, consequently, also in the femur. Thus, $k_{BR}$ is the critical factor determining whether the peak impact force in the femur $F_{Femur}$ exceeds the femoral strength $S_{Femur}$. Let’s define the force to strength ratio (FSR) as $FSR = F_{Femur}/S_{Femur}$, and $k_{BR_{crit}}$ as the critical value of $k_{BR}$ within its range resulting in FSR=1. It follows that for all $k_{{BR}} \le k_{BR_{crit}}$, FSR≥1 is reached, and thus a fracture will occur, while all $k_{{BR}} > k_{BR_{crit}}$ result in FSR<1, and subsequently no fracture will occur.

#### Probability of the fall dynamics

The previous section showed how the critical body resistance spring constant $k_{BR_{crit}}$ that results in a FSR=1, and thus in a fracture, is defined. However, deriving the absolute fracture risk requires the likelihood that a specific fall scenario reflected by $k_{BR}$ occurs. To do so, $k_{BR}$ is modelled as a random variable following an exponential probability distribution of the form $f(y) = (\alpha + 1)*y^{\alpha }$. We chose an exponential distribution because of its inherent non-linearity, monotony and simplicity, as it requires only one parameter to be characterised. With a positive α, the function is monotonically increasing in a non-linear way, and the distribution’s shape eventually reflects what can be observed in reality: Only around 1–3 % of all falls result in a hip fracture (Yang et al. 2020; Komisar and Robinovitch 2021), indicating that falls with an impact force high enough to cause a fracture are relatively rare. Falls with an impact force below the critical force level are more frequent and occur with a higher probability.

To assign $k_{BR}$ to the probability distribution, the relative $k_{BR}$ given as $rk_{BR} = k_{BR}/k_{BR_{max}}$ was calculated, so that $rk_{BR} \in [0,1]$. Accordingly, the probability density function describing the occurrence of a fall resulting in a specific impact velocity is given by

5 $$\begin{aligned} f(y = rk_{BR}) = P(y) = (\alpha + 1) * y^\alpha \end{aligned}$$

and the cumulative distribution function is given by

6 $$\begin{aligned} F(y=rk_{BR}) = P(Y \le y) = y^{(\alpha + 1)} \end{aligned}$$

Since all $k_{{BR}} \le k_{BR_{crit}}$ result in a fracture, it follows that the probability of a fracture conditioned on a fall $p_S$ can be derived by evaluating Equation (6) for the relative $rk_{BR_{crit}}$, given by

7 $$\begin{aligned} p_S = F(y = rk_{BR_{crit}}) \end{aligned}$$

### Model parameters

#### Personalised parameters

The derivation of the personalised fall rate λ with the model developed in previous work requires the number of falls during the past 12 months as an input variable (Wapp et al. 2024). Next to height and weight, the personalised parameters required for the impact force model can be extracted from QCT images. These include femoral height $h_{Femur}$, hemi-pelvis width $h_{HP}$, soft tissue thickness $h_{ST}$, femoral strength $S_{Femur}$ and femoral stiffness $k_{Femur}$ (Table 2). Ultimate strength and stiffness of the femur can be estimated using FEA, which normally requires a BMD calibration. The various modelling options when performing FEA may require the use of correction factors or cross-calibrations between specific modelling approaches. In particular, a correction may be applied for the strain rate-dependent behaviour of bone tissue, which is not accounted for in commonly used quasi-static models. The processing of the computed tomography (CT) images and the FEA pipeline used in this work, as well as the exact definition of the extracted parameters, is presented in 2.3.2.

In case no QCT image is available, personalised parameters may be estimated through established relationships with other clinical variables. For example, bone strength could be substituted with femoral aBMD obtained from DXA (Bouxsein et al. 2020).

**Table 2:** Overview of the personalised parameters

Parameter	Unit	Source	Substitution equations
Fall rate λ	Falls/Year	Derived with previously developed models; requires the number of falls in the previous year as input variable (Wapp et al. 2024)	–
Height	[m]	needs to be measured	–
Weight	[kg]	needs to be measured	–
Soft tissue thickness $h_{ST}$	[m]	from algorithm based on femur coordinate system	Using BMI and sex
			$$h_{ST_{female}} = -8.481 + 2.387 * BMI$$
			$$h_{ST_{male}} = -27.852 + 2.6 * BMI$$
Femoral height $h_{Femur}$	[m]	from femur coordinate system (Dudle et al. 2023)	Using height
			$$h_{Femur} = -0.011805 + 0.044875*height$$
Hemi-pelvis width $h_{HP}$	[m]	from femur coordinate system (Dudle et al. 2023)	Using sex, height, and $h_{Femur}$
			$$h_{HP_{female}} = (0.5 * 0.219 * height) - h_{Femur}$$
			$$h_{HP_{male}} = (0.5 * 0.200 * height) - h_{Femur}$$
Femoral strength $S_{Femur}$	[N]	from QCT based FEA (Dudle et al. 2023)	Using total hip aBMD
			$$\log (S_{Femur}) = 8.53751 + 1.56982 * \log (aBMD)$$
Femoral stiffness $k_{Femur}$	[N/m]	from QCT based FEA (Dudle et al. 2023)	Using aBMD-derived femoral strength
			$$k_{Femur} = 146400 + 353.4 * S_{Femur}$$

The substitution equations for $S_{Femur}$ and $k_{Femur}$ calculate the quasi-static values

#### Fixed parameters

The parameters for the standard non-linear solid characterising the soft tissues were fitted using data from indentation experiments, which investigated the intrinsic, non-linear visco-elastic behaviour of excised human trochanteric soft tissue samples. The elastic and damping properties were assumed to be universal and independent of the soft tissue thickness $h_{ST}$. Consequently, the parameters that constitute the standard non-linear solid, $fas_{S0}$, $fas_{S1}$ and $\mu _{D1}$ are only personalised in terms of thickness. The detailed description of the material model and the derivation of these parameters are described in the supplementary materials.

Due to the lack of a method to compute a personalised hemi-pelvis stiffness, $k_{HP}$ was defined as the median of the quasi-static stiffness values reported in an experiment testing 10 isolated pelvis bones in a sideways configuration (Guillemot 1998). We are not aware of any relationship to calculate the pelvis stiffness using other body parameters. Hence, no substitution equation can be provided.

Ground stiffness $k_{G}$ was calculated as structural stiffness for an axial load case, which is given by $k_{G} = E_{G} \, \frac{R_{impact}^2 \pi }{h_{G}}$ with $E_{G}$ as the Young’s modulus, $h_{G}$ as the height of the ground, and $R_{impact}$ as the radius of the contact area upon impact, which is assumed to be circular. $R_{impact}$ was set to 2.5 cm, which is in line with different studies that investigated forces and contact areas upon impact on the hip (Levine and Laing 2017; Levine et al. 2018; Pretty et al. 2021).

Furthermore, a strain rate correction factor of 1.369 was applied to all quasi-static derived parameters, which include the femoral strength $S_{Femur}$ and the stiffness values of the femur $k_{Femur}$ and the hemi-pelvis $k_{HP}$. The factor compensates for the higher strain rate occurring during a fall on the hip and was derived from both experimental and analytical data. The detailed derivation can be found in the supplementary material. An overview of all fixed parameters is given in Table 3.

**Table 3:** Overview of the fixed parameters

Parameter	Unit	Value	Source
$fas_{S0}$	[N·m$^2$]	0.0133	From indentation experiments
$fas_{S1}$	[N·m$^2$]	0.2518	From indentation experiments
$\mu _{D1}$	[N·m$^2$ ·s]	0.0063	From indentation experiments
$k_{HP}$	[N/m]	587’000	Median value from isolated pelvis experiments (Guillemot 1998)
$h_{G}$	[m]	0.05	
$E_{G}$	[Pa]	$3\times 10^{10}$	Elastic modulus of concrete
$R_{impact}$	[m]	0.025	Estimated based on data from literature (Levine and Laing 2017; Levine et al. 2018; Pretty et al. 2021)
$k_{G}$	[N/m]	$7.854 \times 10^{10}$	Structural stiffness, calculated with $k_{G} = E_{G} \, \frac{R_{impact}^2 \pi }{h_{G}}$
Strain rate correction factor	[–]	1.369	Derived with the relationship described by Peruzzi et al. (2021)

$k_{HP}$ refers to the quasi-static value

### Data processing and model calibration

We use data from the AFFIRM-CT cohort to illustrate the processing of clinical data for model application and the calibration of the model for a specific cohort or population. Furthermore, some of the equations we suggest for substituting missing QCT-based parameters have been derived using this dataset. Additionally, the ranges of the personalised parameters that are used for the sensitivity analysis are derived from this cohort.

#### Data

The AFFIRM-CT study ("A Fragility Fracture Integrative Risk Model for CT Recycling") is a prospective observational study that was conducted with the aim of identifying factors for fall and fragility fracture risk prediction (https://doi.org/10.1186/ISRCTN72750412). Briefly, community-dwelling older adults aged 65 and older who underwent a CT scan that included the hip at the University Hospitals of Bern and Geneva were eligible for study participation. Exclusion criteria were a prior hip fracture, life expectancy of less than one year, being bedridden or in a wheelchair, living in a nursing home, suffering from a bone disease, or cognitive impairment. Eligible individuals were contacted by phone or letter for enrolment in the study. Enrolled participants were invited for a single visit to the respective medical centre. During the visit, participants underwent DXA scans (Bern: Hologic Discovery A, Geneva: Hologic Discovery W) of the lumbar spine and hip. At the same time, various variables in the field of medical history, cognitive status and physical performance were assessed. Participants were enrolled and examined between 2021 and 2024 and are being followed for 36 months via phone calls to assess incident falls and fractures. The last participants are expected to complete follow-up in June 2026. The study was approved by the local ethics commissions in Bern and Geneva (BASEC ID: 2019–01,327). All participants provided written informed consent.

#### CT processing and FEA

CT processing and FEA were performed using existing methods from our group (Dudle et al. 2023, 2024). Briefly, after calibration and segmentation, an implicit coordinate system of the proximal femur consisting of two intersecting axes (neck and proximal shaft axis) was introduced. After a rigid body transformation of the segmentation mask, a voxel mesh file is written for a load case mimicking a standardised fall to the side (diaphysis axis inclined by 10$^\circ$ with respect to the ground). A non-linear quasi-static finite element (FE) solver was used with an elastic-perfectly plastic constitutive model. A load is applied, and femoral strength is defined as the reaction force acting on the femoral head when it reaches a vertical displacement of 4% of the vertical distance between the femoral head centre and the most lateral point of the greater trochanter in the fall configuration. The same distance is used for the femoral height $h_{FH}$ in the mechanical model. The knowledge of the two femoral head centres is used to estimate the personalised pelvic width, from which the hemi-pelvis width $h_{HP}$ is derived. Trochanteric soft tissue thickness $h_{ST}$ was measured with an algorithm based on the same femoral coordinate system, finding the shortest distance from the most lateral point of the greater trochanter in fall configuration to the air-soft-tissue boundary. A brief description of the algorithm can be found in the supplementary materials. Since the femur is represented by a linear spring in the mechanical model, femoral stiffness $k_{Femur}$ was calculated as secant stiffness. Femoral strength, as well as femoral and pelvis stiffness, were corrected for strain rate dependence using the correction factor given in Table 3. More details on the CT processing and FEA can be found in the supplementary material.

#### Substitution equations for QCT-based parameters

In case some of the personalised QCT-based parameters are not available, these values could be derived using other parameters. Here, substitution equations for femoral height $h_{Femur}$, femoral strength $S_{Femur}$ and femoral stiffness $k_{Femur}$ were derived with the AFFIRM-CT data. Femoral height $h_{Femur}$ can be estimated using an individual’s overall height. Femoral strength $S_{Femur}$ is correlated with total hip aBMD from DXA, and femoral stiffness $k_{Femur}$ is correlated with femoral strength. Trochanteric soft tissue thickness $h_{ST}$ can be derived with BMI and sex. The detailed derivation of the substitution equations with the AFFIRM-CT data is provided in the supplementary materials.

Hemi-pelvis width $h_{HP}$ can be derived using the relationship presented by Contini (1972), calculating hip width given height and sex. As this relationship calculates the total hip width, the femoral height was subtracted. Table 2 includes an overview of the personalised model parameters and their substitution equations.

Thus, the minimal set of required parameters to substitute all QCT-based input parameters includes sex, height, weight and total hip aBMD obtained from DXA.

#### Model calibration

The first step of model calibration was calculating $k_{BR_{crit}}$ for every participant. If both sides of the femur could be computed, the side-dependent parameters ($S_{Femur}$, $k_{Femur}$, $h_{Femur}$, $h_{ST}$) were taken from the side with the smaller femoral strength. If only one side was computable, those values were used. If the soft tissue thickness $h_{ST}$ was not detectable on the weaker strength side but was available on the other side of the body, that value was used. All missing variables were substituted with the regression equations presented in Table 2. Observations with a follow-up time of less than 6 months were excluded. Because prior falls were only recorded for the 6 months preceding the examination, the total number of prior falls was calculated by summing the number of falls reported during the 6 months before the examination and those prospectively reported during the first 6 months of follow-up.

The critical value of the body resistance spring constant $k_{BR_{crit}}$ was derived by solving the DAE system (see Equation (2)) so that the $FSR = F_{Femur} / S_{Femur} =1$, with a tolerance level of 1%. To calibrate the model, the parameter α characterising the exponential function (see Equation (6)) was fitted so that the predicted fracture incidence of the AFFIRM-CT cohort corresponded to the annual age-adjusted hip fracture incidence in Switzerland (men: 160 per 100’000 per person years, women: 400 per 100’000 per person years) (Lippuner et al. 2022). The incidence was weighted according to the share of male and female participants in the analysed data, resulting in an incidence of 238 per 100’000 person years.

### Sensitivity analysis

The goal of the sensitivity analysis was to assess the influence of the different parameters of the calculator on the fracture risk. Data from the AFFIRM-CT cohort was used to derive the mean and standard deviations (SD) for all required parameters. Due to the lack of information about the pelvis stiffness, these numbers were calculated from the data presented by (Guillemot 1998). SD were calculated for all variables except the fall rate. A parameter’s influence on the fracture risk was examined by computing the conditional fracture probability $p_S$ and the one-year fracture probability $P({\textit{Fracture in} } (0, T=1])$ for SD between -2 and 2 while holding all other parameters at the mean value (SD = 0). For the calculation of the fracture probability, the fall rate was also held at its mean. Furthermore, risk gradients within 1 SD around the reference values were calculated to quantify the influence of a parameter. To assess the influence of decreased bone strength on the sensitivity of the other parameters, the procedure was repeated with all parameters being held at their mean values (SD = 0) except for bone strength, which was set to a constant value of -1 SD below the population’s mean.

As defined in Equation (1), the probability of a fracture is dependent on the fall rate λ and the conditional fracture probability $p_S$. To get insight into the influence of those two parameters on the fracture risk, the risk was calculated for a range of fall rate values and different $p_S$. Furthermore, the influence of the soft tissue thickness $h_{ST}$ on the peak impact force was assessed by evaluating the DAE system (see Equation (2)) when all parameters (inclusive $rk_{BR}$) were held constant at the cohorts’ mean, and varying $h_{ST}$ in its range. A linear regression was then fit to compare the influence of the soft tissue thickness on the peak impact force to the results derived by (Robinovitch et al. 1995). Similarly, the influence of the stiffness parameter $k_{BR}$ on the peak impact forces was evaluated by varying it from its minimum to maximum while holding all other parameters at the mean value.

### Software

The software for the impact force model was implemented in python using the packages pandas and numpy. The DAE system was solved with a solver for dynamic simultaneous simulations (mode 4) provided by the gekko package (Hedengren et al. 2014; Beal et al. 2018).

## Results

In total, 374 participants were enrolled in the AFFIRM-CT study. One individual turned out not to be eligible (prior hip fracture), one individual was lost before the examination (death), and one individual withdrew consent during the follow-up time, and 10 individuals were excluded due to a follow-up time of less than six months, resulting in 361 individuals included in this analysis. Since some QCT images were not successfully processed (e.g., the implicit coordinate system of the femur could not be defined) or did not include the full region of interest (e.g., the soft tissue-air boundary was not in the field of view), several QCT-related variables had missing values (see column "NA’s" in Table 4). These were substituted using the equations presented in Table 2. The majority of the participants were male (n = 244, 67.5 %), and the mean age of the cohort at examination was 73.77 years (SD 6.05). The average BMI was 27.19 (SD 4.89). The summary statistics of the set of minimal required parameters and the QCT derived parameters are presented in Table 4.

**Table 4:** Summary statistics of the model parameters and the output values. The numbers include substituted missing values

Variable	Mean (SD)	Min	Max	NA’s $^a$
*Minimal required parameters*				
Weight [kg]	78.5 (16.9)	39.5	148.5	–
Height [cm]	169.5 (9.0)	139.7	202.0	–
Prior falls	0.34 [0, 0] $^b$	0	13	–
TH aBMD [g/cm$^2$]	0.890 (0.153)	0.525	1.526	3
*QCT derived parameters*				
$h_{ST}$ [mm]	43.21 (17.80)	8.01	110.54	49
$h_{Femur}$ [mm]	64.41 (6.02)	46.75	79.34	50
$h_{HP}$ [mm]	91.31 (8.06)	74.02	131.07	40
$S_{Femur}$ $^{c}$ [N]	4226 (1416)	1704	11’199	50
$k_{Femur}$ $^{c}$ [N/m]	$1.641\times 10^6$ $(5.210\times 10^5)$	$6.497\times 10^5$	$4.444\times 10^6$	50
*Model output*				
Fall rate λ	0.786 [0.594, 0.837] $^b$	0.594	5.035	–
$rk_{BR_{crit}}$ [–]	0.615 (0.146)	0.000	0.908	–
$v_{imp}$ $^d$ [m/s]	2.26 (0.41)	1.12	3.72	–
$p_S$	$3.358 \times 10^{-3}$ [$4.040 \times 10^{-6}, 1.224\times 10^{-3}$] $^b$	0.000	0.151	-
$P(\text {Fracture})$	$2.383 \times 10^{-3}$ [$3.360 \times 10^{-6}, 8.229\times 10^{-4}$] $^b$	0.000	$9.080\times 10^{-2}$	-

$^\textrm{a}$Number of missing values before substitution; $^\textrm{b}$Inter quartile range instead of standard deviation due to non-normal distribution; $^\textrm{c}$Quasi-static values; $^\textrm{d}$ for $k_{BR} = k_{BR_{crit}}$. Abbreviations: SD, standard deviation; NA’s, missing values; TH, total hip

**Fig. 2:**
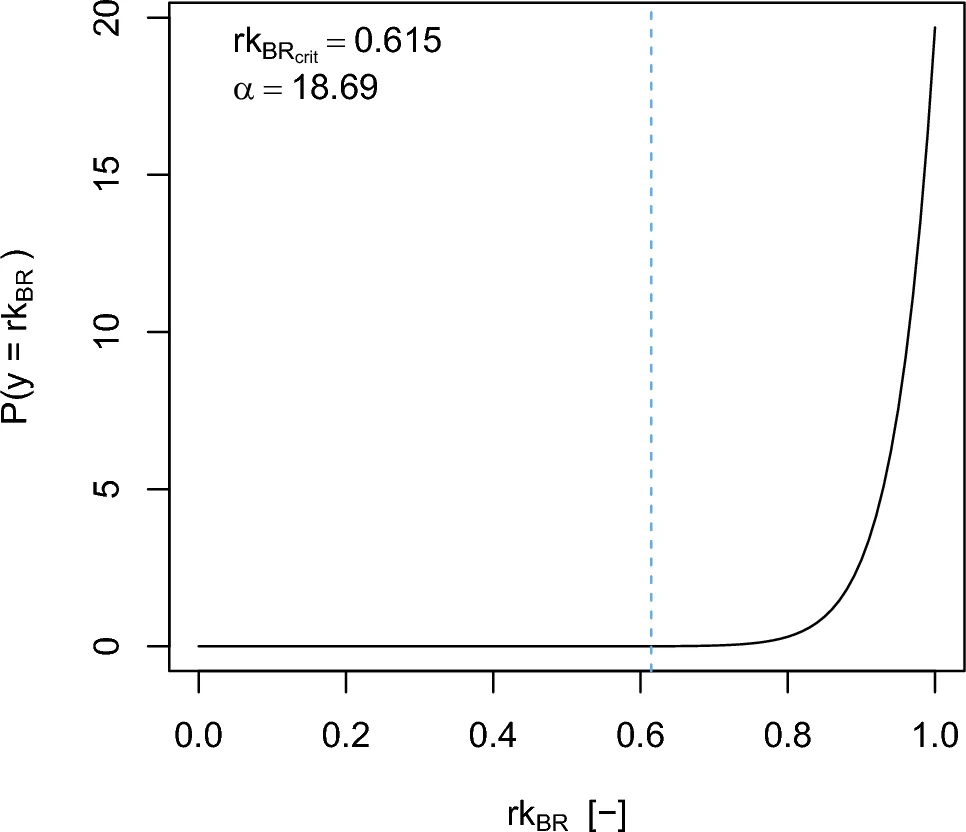
Probability mass function modelling the stochastic aspects of the fall dynamics represented by $$ rk_{BR}$$. The blue line indicates the mean value of $$rk_{BR_{crit}}$$ in the AFFIRM-CT cohort. The exponent α was fitted so that the hip fracture incidence of the cohort corresponds to the age-adjusted annual incidence in Switzerland

The mean value of the critical relative body resistance stiffness $rk_{BR_{crit}}$ was 0.615. The corresponding average impact velocity of the hip with $k_{BR}$ at its critical value was 2.26 m/s. All these values are also presented in Table 4. The parameter α defining the shape of the exponential distribution describing the chances of different fall scenarios was fit to α=18.69. The probability mass function for $rk_{BR}$ describing the chances of varying fall dynamics is presented in Fig. 2. The blue dotted line indicates the cohort’s mean of $rk_{BR_{crit}}$. The integrated area from 0 to 0.615 refers to the conditional fracture probability $p_S$.

The mean of the conditional fracture probability was $p_S=3.358 \times 10^{-3}$, and the mean of the probability of at least one hip fracture $P({\textit{Fracture in} } (0,T=1]) = 2.383 \times 10^{-3}$ (Table 4). This corresponds to the annual fracture incidence in Switzerland for which the model was calibrated (Lippuner et al. 2022).

**Fig. 3:**
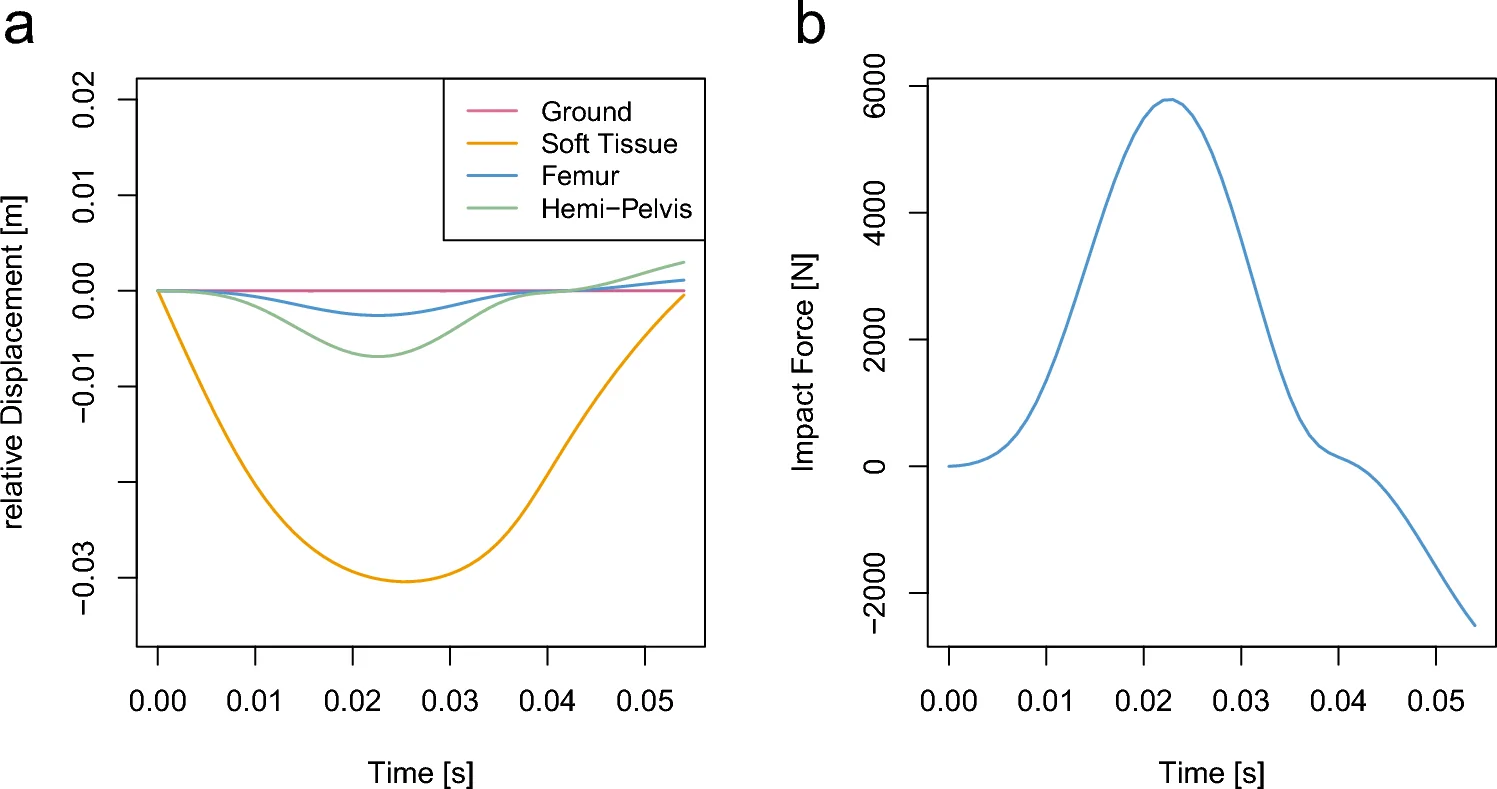
Example plots of (**a**) the relative displacement in every component of the mechanical model upon impact and (**b**) the resulting impact force. The time t=0 is defined as the start of the impact on the ground

In Fig. 3a, an example of the relative displacement during the impact in the elements of the mechanical model is presented. It is clearly visible that the soft tissues deflect the most upon impact. Figure 3b shows an example of the resulting impact force. The example is calculated with the AFFIRM-CT data’s mean values for all parameters.

**Fig. 4:**
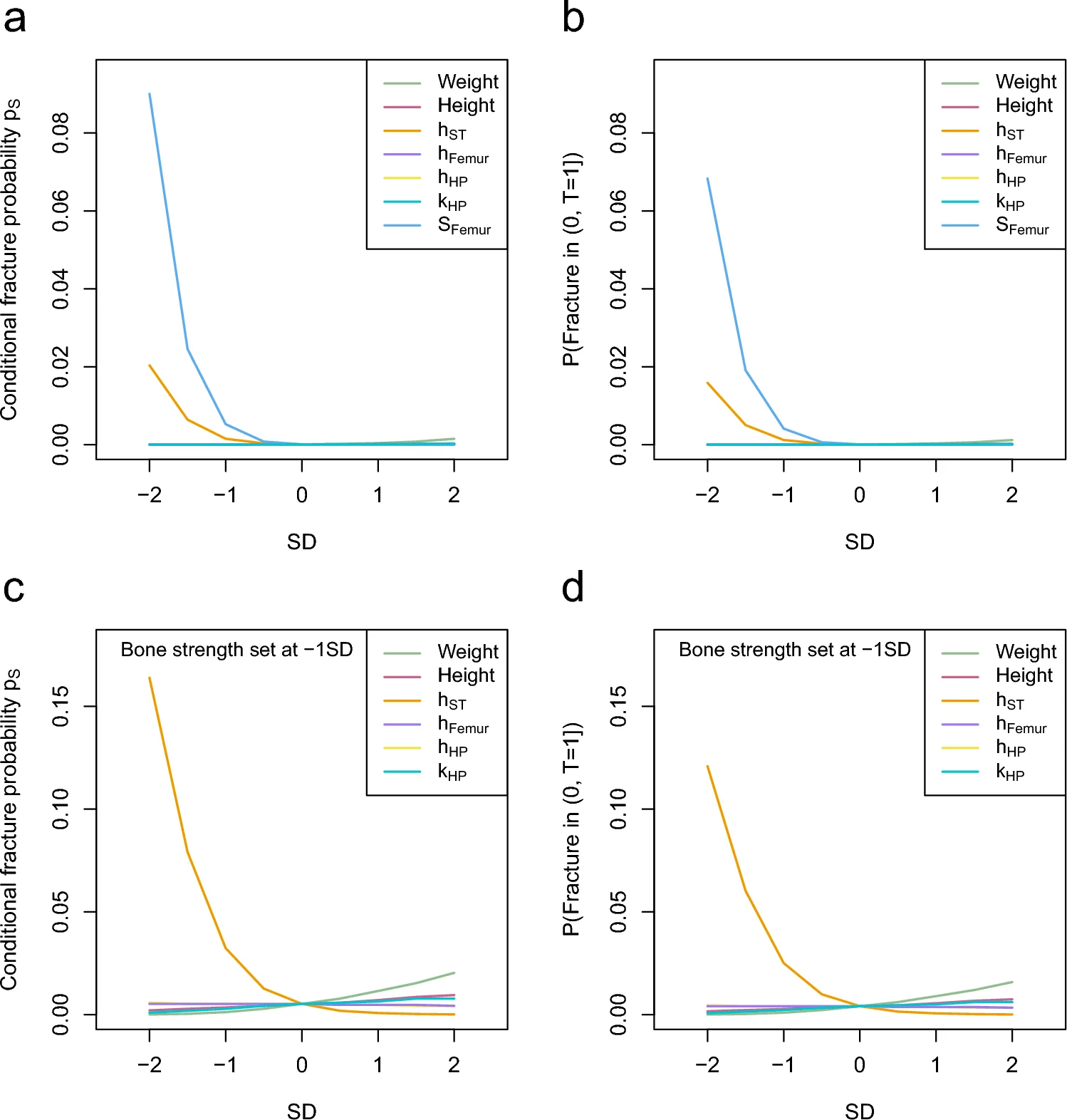
Results from the sensitivity analysis showing the influence of the model parameters on the (left column) probability of a fracture conditioned on a fall $p_S$, and (right column) on the probability of at least one fracture in the time period of one year $P({\textit{Fracture in} } (0,T=1])$. Plots a and b show the influence of every parameter when all others are held at the population mean (SD = 0). Plots c and d show the influence with all parameters held at their mean values except for bone strength, which was set to a constant value of -1 SD below the population mean (2810 N)

**Fig. 5:**
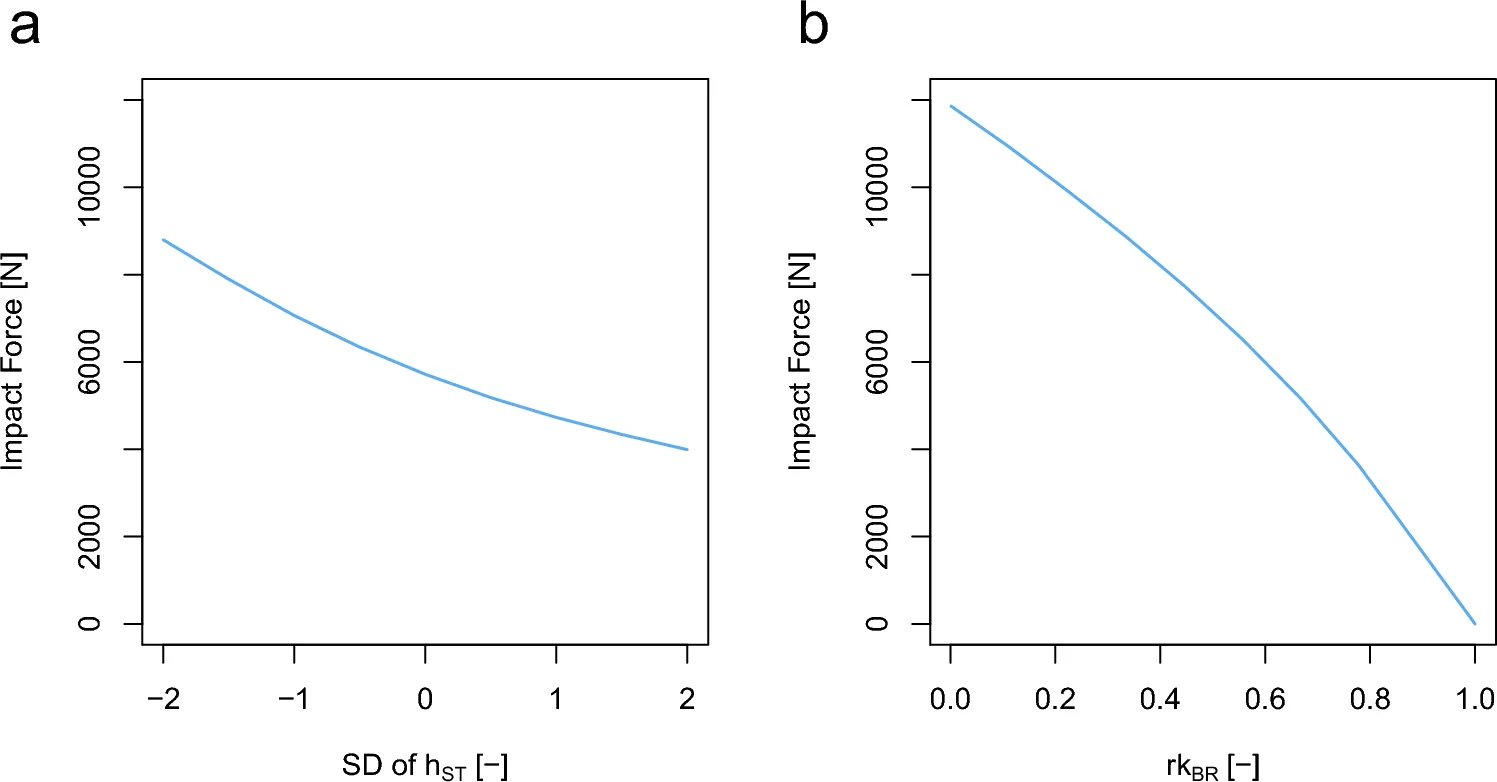
Influence of (**a**) $h_{ST}$ and (**b**) $rk_{BR}$ on the peak impact force in the hip. The displayed forces are strain rate corrected

Figure 4 shows the influence of the different parameters on the conditional fracture probability $p_S$, and the fracture risk $P({\textit{Fracture in} } (0,T])$. Figure 4a and b present the results of the influence of every parameter when all others are held at the population mean (SD = 0). Thereby, the femoral strength $S_{Femur}$ and the soft tissue thickness $h_{ST}$ are the two dominant factors, increasing the fracture risk with decreasing values. Increasing weight resulted in a slight increase in fracture risk, too. In Fig. 4c and d, all parameters were held at their mean values (SD = 0) except for bone strength, which was set to -1 SD (2810 N) below the population mean. It can be observed that the overall fracture risk is substantially higher when bone strength is decreased ( $P({\textit{Fracture in} } (0,T]) = 5.968 \times 10^{-5}$ for all values set to SD = 0; $P({\textit{Fracture in} } (0,T]) = 4.115 \times 10^{-3}$ when bone strength is set to -1 SD). Furthermore, the influence of most parameters on the fracture risk changed slightly and became stronger. The soft tissue not only resulted in an increased fracture risk with decreasing thickness but also appeared to be protective with increasing thickness. Similarly, an increase in weight led to a higher fracture risk, while a decrease in weight had the opposite effect. These findings were also reflected in the results of the fracture risk gradients (Table 5). For the analysis of the influence of the parameters when all others were held at the mean values, the gradients of femoral strength soft tissue thickness were around one order of magnitude larger (Column 1). Furthermore, the gradients of all parameters were increased by at least one order of magnitude for the analysis with a decreased bone strength (Column 2), indicating that the influence of the parameters on the fracture risk is dependent on bone strength.

**Table 5:** Fracture risk gradients of the model parameters quantifying the influence of every parameter when all others are held at the mean (SD = 0) (Column 1), or with all parameters held at their mean values except for bone strength, which was set to a constant value of -1 SD below the population mean (2810 N; Column 2)

	1	2
*Variable*		
Weight	$1.407 \times 10^{-4}$	$3.870 \times 10^{-3}$
Height	$3.001 \times 10^{-5}$	$1.184 \times 10^{-3}$
$h_{ST}$	$-2.350 \times 10^{-4}$	$-8.444 \times 10^{-3}$
$h_{Femur}$	$-8.391 \times 10^{-9}$	$-3.931 \times 10^{-4}$
$h_{HP}$	$-1.125 \times 10^{-8}$	$-3.932 \times 10^{-4}$
$k_{HP}$	$6.147 \times 10^{-5}$	$1.182 \times 10^{-3}$
$S_{Femur}$	$-6.175 \times 10^{-4}$	$-1.848 \times 10^{-2}$

The influence of the soft tissue thickness $h_{ST}$ on the peak impact force is shown in Fig. 5a. Thereby, the peak impact force increases with decreasing soft tissue thickness in a non-linear relationship. The linear regression fitted to these data points reveals a decrease of −66.89 N per every additional mm of soft tissue thickness (p-value = 7.76e−07) in peak impact force.

In Fig. 5b, it is shown how $k_{BR}$ influences the peak impact force through its energy-storing mechanism. The minimum peak impact force of 0 N is reached when all energy is absorbed during the fall, reflected by $rk_{BR_{crit}}=k_{BR_{max}}$. On the contrary, when no energy is absorbed during the descent, represented by $rk_{BR_{crit}}=0$, the peak impact force is at its maximum (here 11’858 N). This example was calculated with all parameters set to the cohort’s mean values.

**Fig. 6:**
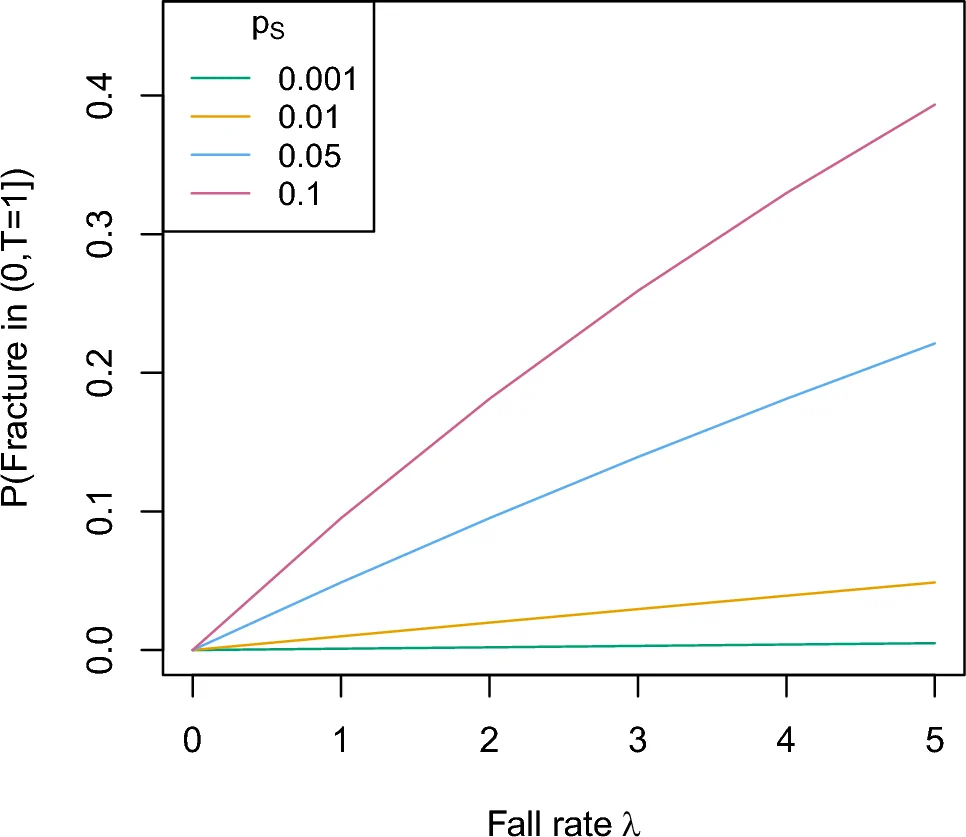
Influence of the fall rate λ and the conditional fracture probability $p_S$ on the fracture risk

Figure 6 illustrates the influence of the fall rate λ and the conditional fracture probability $p_S$ on the absolute fracture risk. It is clearly visible that the risk of a fracture increases with an increasing fall rate λ and an increasing conditional fracture probability $p_S$ in a non-linear manner.

## Discussion

In this article, we present a new mechanistic stochastic fragility hip fracture risk calculator that is based on mechanistic and stochastic modelling approaches. The calculator integrates the stochastic aspects of falls and the fall-induced impact force and uses bone strength estimate from QCT FEA to calculate a one-year absolute fracture risk. In contrast to other mechanistic prediction tools, the mechanical model presented here is composed of rheological elements representing the trochanteric soft tissue, the femur, the pelvis and the ground, allowing the evaluation of the influence of the different elements on the fracture risk. Furthermore, no specific fall scenarios are modelled, but the range of possible impact forces given the anthropometric measures for an individual is calculated. An exponential probability distribution is then used to derive the probability of an impact force high enough to initiate a fracture to occur. By integrating this probability with the probability of at least one fall, an absolute fracture risk is derived. All input variables, except for height, weight, and the number of falls in the past year, can be extracted from the QCT image. In case such images are not available, substitution equations to calculate the required input parameters based on anthropometric and densitometric data are provided.

Comparing the output variables of the mechanical model with the literature, these are quite consistent with experimentally derived values. The average impact velocity for $k_{BR_{crit}}$ is 2.27 m/s (range 1.12–3.72 m/s). This is comparable with experimental values measured during unintentional sideways falls of young, healthy volunteers, reporting a mean velocity of 2.75 m/s (SD 0.42 m/s) (van den Kroonenberg et al. 1996) and 3 m/s (SD 0.83 m/s) (Feldman and Robinovitch 2007). The models’ predicted impact forces, given the fall dynamics, range from 0 to 11’791 N when all parameters are held at the cohort’s mean value. Robinovitch et al. reported peak impact forces between 4050–6420 N measured with an impact pendulum (Robinovitch et al. 1995), and Fleps et al. derived impact forces between 2947–7601 N using a sideways fall simulator (Fleps et al. 2019). Thus, the impact forces derived from the model presented here cover a wider range when compared to experimentally derived values. However, this was to be expected, as the full range of $k_{BR}$, representing all fall scenarios from no energy storage at all to complete energy absorption during the descent, was modelled. The regression equation that quantifies the influence of the soft tissue thickness on the peak impact force is close to what was reported by Robinovitch et al. (1995) (model here: - 66.89 N/mm; Robinovitch et al.: - 71 N/mm). This indicates that the standard non-linear solid captures the non-linear visco-elastic behaviour of the trochanteric soft tissue during an impact on the hip.

The sensitivity analysis showed that bone strength, soft tissue thickness, and fall rate are the key parameters that determine the fracture risk. Decreased bone strength and/or low soft tissue thickness result in a high fracture probability; expectedly, the influence of these two parameters on fracture risk is even more pronounced in individuals with both decreased bone strength and low soft tissue thickness. At the same time, increased soft tissue thickness or low body weight in individuals with decreased bone strength can be protective of fragility fractures. The thinned Poisson process scales the conditional fracture probability $p_S$ by combining it with the fall rate λ and, through that, calculates an absolute fracture risk. Fracture risk increases with an increasing fall rate, but its influence is also dependent on the conditional fracture probability $p_S$ itself; the absolute risk is low if the conditional probability is low, but rises in a non-linear way if this number is increasing. It shows clearly that both a high conditional fracture probability $p_S$ (e.g., due to decreased bone strength, low soft tissue thickness, or the inability to dampen a fall), together with a high fall rate, are required for a fracture to occur. The exposure to either a high conditional fracture probability or a high fall frequency might be insufficient to initiate a fracture. Hence, the model eventually gives insight into why aBMD alone, known for its low sensitivity and high specificity (Hernlund et al. 2013; Marshall et al. 1996; Kanis et al. 2002), is not a robust predictor for hip fractures, and why many fractures happen in individuals in the normal or osteopenic range (Mai et al. 2019; Cranney et al. 2007; Siris et al. 2004): the overall fracture risk appears to be fall rate dependent. In line with these findings, several studies have demonstrated the predictive ability of falls for fragility fractures (Masud et al. 2011; Harvey et al. 2018). Thus, the model seems to capture the relevant aspects of the risk of fragility hip fractures observed in empirical data and eventually is able to explain why individuals with identical bone strength can have different fracture outcomes.

Our model might be criticised for various assumptions and simplifications. First, we used a simple mass-spring model to calculate the impact velocity that neglects the biomechanics of a fall. However, although it is possible to model fall scenarios with dynamic models, the countless possibilities of how a fall could occur raise the question: which fall scenarios should be modelled? Due to the limited information to answer these questions, we decided to model the fall dynamics and influence of pre-impact movement strategies on the impact velocity indirectly by deriving the range of physically possible impact velocities given an individual’s anthropometric parameters. Therefore, aspects that vary from fall to fall and that can not be predicted, such as the initial height and fall direction, the presence of rotational movement, different impact orientations, or the outstretching of the hand, are represented by the range of $k_{BR}$.

Next, we acknowledge that the use of a 1D mechanical model to simulate an impact on the hip following a sideways fall is a simplification of reality. In addition, the model lacks elements representing the skin, cartilage, and ligaments of the hip complex. And last, the computation of bone strength in just one loading configuration could be criticised. Nonetheless, we believe that modelling different impact orientations of the hip is a complex problem that again relies on many assumptions and simplifications, e.g. the variation of the soft tissue thickness given impact orientation or the lack of knowledge about the anisotropic material behaviour of the tissues in the hip complex. Similarly, the computation of bone strength for different impact orientations with an FEA pipeline that was validated with experimental data of one loading case eventually introduces an undefined error in the strength estimation due to the anisotropy of the bone tissue. Based on these considerations, we have decided to model the impact on the hip with a simplified 1D mechanical model. The influence of factors that affect the visco-elastic behaviour of the hip and that can vary among different fall scenarios (e.g. the impact orientation or muscle activation) was again indirectly modelled with the fall dynamics, represented by the range of $k_{BR}$.

The use of a stochastic distribution to assign a probability to different fall-induced impact forces opens several opportunities but also has its limitations. On the one hand, it allows the calculation of an absolute fracture risk within a mechanistic setting. Although mechanistic models can precisely estimate the outcome of an event (here, the impact force following a fall), they do not directly inform about the chances of this happening. To derive a risk, the output needs either to be analysed with a statistical regression model (e.g. by relating the force-to-strength ratio to the fracture risk using a Cox proportional hazard model Dufour et al. 2012) or a stochastic distribution is needed to describe the occurrence of an input variable of the model. To our knowledge, there exists only one other mechanistic model following the latter approach (Bhattacharya et al. 2019). In this model, the impact force is derived by modelling a sideways fall with an inverted pendulum, and stochastic distributions are used to assign the probability of possible angles of the inverted pendulum, characterising different scenarios of a sideways fall.

However, the assumption about the shape of such a probability distribution is a major limitation. We chose an exponential distribution because its shape eventually reflects what is observed in clinical data: Only around 1–3% of all fall events are severe enough to result in a fracture (Yang et al. 2020; Komisar and Robinovitch 2021); falls resulting in an impact force below the critical level, here defined through $k_{BR}>k_{BR_{crit}}$, are more likely to occur. With α=18.69, low values of $k_{BR}$ are highly unlikely, while the probability of $k_{BR}>0.8$, which represents impact forces in the lower range, increases exponentially. In addition, the exponential distribution has the advantages of being characterised by only one parameter and its inherent non-linearity.

Both the choice of the stochastic distribution and its parameter α have a direct influence on the calculated fracture risk. The direct validation of whether these assumptions are appropriate is challenging, as the collection of the required empirical data is not feasible. Nevertheless, this can be done indirectly by evaluating the model’s prediction accuracy with a receiver operating characteristic curve in a cohort with sufficient statistical power. Here, we have fitted α indirectly so that the fracture incidence in the AFFIRM-CT data set corresponds to the age-adjusted fracture incidence in Switzerland. In order to apply the model to other countries or geographical regions, α would have to be fitted with the respective incidences. Furthermore, since the ability to attenuate a fall may differ between men and women, α could be fitted separately for each sex. Following an even more personalised approach, the distributions could be adjusted based on an individual’s physical ability to dampen a fall. Thereby, α could be defined by a function that depends on age, sex, physical performance tests and other factors that reflect this ability.

A strength of the presented model is the selection of the required input parameters. For individuals with an available QCT image, the only additional parameters required are weight, height and the number of falls within the prior year. Thus, the model is well-suited for application in an opportunistic screening setting. If no QCT image is available, these parameters can be estimated using established relationships with other clinical variables or alternative methods measuring the variable of interest. Here, the suggested substitution equations require only height, weight, sex, and total hip aBMD obtained from DXA as variables. Thus, with the exception of sex used in two substitution equations, the model is solely based on physical parameters. Accordingly, it is not necessary to consider the epidemiological variation of risk factors by sex, age or ethnicity. In fact, these factors affect the mechanical parameters in a complex way. To ensure that the suggested substitution equations apply to data other than the AFFIRM-CT data, they must be validated using other datasets. At the same time, the influence of the error introduced by substitution equations on the fracture risk must be assessed thoroughly.

Due to the model’s mechanistic nature, different aspects of fracture risk prediction and prevention strategies could be assessed once the model is validated. First, it could give insights into whether improved femoral bone strength estimates, e.g. from high-resolution peripheral quantitative computed tomography (HR-pQCT), in comparison to aBMD or QCT-FEA based bone strength, can improve fracture risk prediction. Thereby, it could be assessed whether the precision errors of the respective methods have a relevant influence on the change in fracture risk. Furthermore, the effect of changes in aBMD upon osteoporosis treatment on the fracture risk could be quantified. In addition, the model allows the assessment of the protective effect of an individual wearing hip protectors as suggested by Robinovitch et al. (1995), Laing and Robinovitch (2010) by adding it as an additional rheological element into the mechanical model. And last, the model could be used to quantify the effect of different flooring materials on the fracture risk, as already investigated by Laing et al. (2006).

Several aspects of the calculator could be improved and further developed. Due to the lack of a method to estimate a personalised pelvis stiffness, we assumed a constant value based on data from literature (Guillemot 1998). The mechanical model could be improved with a method to extract a personalised pelvis stiffness from QCT images. At the same time, the results from the sensitivity analysis indicate that pelvis stiffness has a limited influence on the fracture risk. Furthermore, instead of modelling the femur with a linear spring, the non-linear force-displacement curve of the FEA could be integrated to derive the peak impact force. Another aspect is the time horizon of prediction. For the analysis of the data presented here, a time span of T = 1 was chosen to predict a one-year fracture risk. However, by adjusting T to the time interval of interest, the model allows the risk prediction for any time horizon. To do so, variables that are known to change over time, such as the bone strength or fall rate, would need to be adjusted accordingly. This would allow a comparison with FRAX®  that predicts a 10-year fracture probability.

As a next step, future work should validate the model with clinical data and compare the results with those of other available models to assess its predictive ability at the individual patient level.

## Conclusion

This article presents the framework of a novel mechanistic and stochastic fragility hip fracture risk calculator, integrating the mechanical aspects of an impact on the hip together with the stochastic aspects of a fall. The model’s output variables, such as the impact velocity of the hip or peak impact force in the hip complex, align well with experimental data from the literature. Furthermore, the sensitivity analysis showed that the fall rate, trochanteric soft tissue thickness and bone strength are the key parameters that determine the risk of fragility hip fractures. Thus, the model is able to reflect observations from empirical data, indicating that it can capture the intrinsic aspects that affect the risk of fragility hip fractures. The presented model was built in a way that tries to separate the fall-related stochastic aspects from the mechanical aspects of the mechanism causing hip fractures. This allowed for the introduction of a standardised mechanical model, while the stochastic elements were concentrated in the probability distribution describing the fall dynamics. In addition, its mechanistic nature allows the exploration of different aspects that are relevant in fracture risk prediction and prevention, such as the effect of improved bone strength estimates on the prediction accuracy, the influence of drug treatment on the fracture risk, or the preventive effect of hip pads and fall prevention programs. To bring the model one step closer towards clinical applicability, an extensive analysis of its predictive performance for the individual using cohorts with sufficient statistical power will be required to explore its validity.

## Supplementary Information

Below is the link to the electronic supplementary material.

Supplementary file 1 (pdf 355 KB)

## Data Availability

Data will be made publicly available on Zenodo once the follow-up time of the clinical study is finished (June 2026). Until then, the datasets used and analysed in this article are available from the corresponding author upon reasonable request.

## Abbreviations

aBMD: Areal bone mineral density
BMD: Bone mineral density
BMI: Body mass index
CT: Computed tomography
DAE: Differential algebraic equations
DXA: Dual energy X-ray absorptiometry
FE: Finite element
FEA: Finite element analysis
FSR: Force to strength ratio
G: Ground
HP: Hemi-pelvis
HR-pQCT: High-resolution peripheral quantitative computed tomography
QCT: Quantitative computed tomography
SD: Standard deviations
ST: Soft tissue
