# Supplementary material for: A simple personalised prediction model for hip fracture risk based on mechanistic parameters
Source: Biomech Model Mechanobiol. 2026 Mar 30;25(2):36. doi: 10.1007/s10237-026-02051-z (PMC13675280; doi:10.1007/s10237-026-02051-z)
Supplement: Supplementary file 1 [file 10237_2026_2051_MOESM1_ESM.pdf]

Supplementary Materials

to the article

"A simple personalised prediction model for hip fracture risk based on mechanistic parameters"

published in *"Biomechanics and Modeling in Mechanobiology"*

Christina Wapp

August 2025

1 Derivation of the formulas in the Schechner publication

These fromulas have been published in the article "A Poisson process model for hip fracture risk" by Schechner et al., 2010.

The probability of k falls occurring in a specific time period $(0, T]$ is modelled with a Poisson process given by

$$P(k\ falls\ in\ (0,T]) = \frac{e^{-\lambda T}(\lambda T)^k}{k!}$$

with k as the number of falls and λ as the rate parameter characterising the Poisson process. Since a fracture event is only possible once a fall occurs, the measure of interest is the probability of at least one fall, given by

$$P(k \geq 1 \text{ fall in } (0,T]) = 1 - e^{-\lambda T}$$

The probability that the assigned load is bigger than the femoral strength can be described as the conditional probability of a fracture given as a fall and is given by $p_S = P(Fracture|Fall) = P(Load > Strength)$ and is modelled as a random variable following a stochastic distribution. By combining the fall rate parameter λ with the conditional probability of a fracture p_S, a thinned Poisson process given by

$$P(Fracture\ in\ (0,T]) = 1 - e^{-\lambda p_s T}$$

2 Detailed description of the 1D mechanical model formulas

2.1 Definition of the forces in the 1D mechanical model

The forces of the mechanical model are defined as follows:

$$F_{BR} = k_{BR} * (x_4 - cmh - h_G)$$

$$F_{HP} = k_{HP} * (x_4 - x_3 - h_{HP})$$

$$F_{Femur} = k_{Femur} * (x_3 - x_2 - h_{Femur})$$

$$F_{S0} = \frac{fas_{S0}}{h_{ST}^2} * \log\left(\frac{(x_2 - x_1)}{h_{ST}}\right) + 100 * \frac{fas_{S0}}{h_{ST}^2} * \log\left(\frac{(x_2 - x_1)}{h_{ST}}\right)^3$$

$$F_{S1} = \frac{fas_{S1}}{h_{ST}^2} * \left(\log\left(\frac{(x_2 - x_1)}{h_{ST}}\right) - \log\left(\frac{(x_{21} - x_1)}{h_{ST}}\right)\right) + 100 * \frac{fas_{S1}}{h_{ST}^2} * \left(\log\left(\frac{(x_2 - x_1)}{h_{ST}}\right) - \log\left(\frac{(x_{21} - x_1)}{h_{ST}}\right)\right)^3$$

$$F_{D1} = \frac{fas_{D1}}{h_{ST}^2} * \frac{(x'_{21} - x'_1)}{(x_{21} - x_1)} + 100 * \frac{fas_{D1}}{h_{ST}^2} * \frac{(x'_{21} - x'_1)}{(x_{21} - x_1)} * 3 * \log\left(\frac{(x_{21} - x_1)}{h_{ST}}\right)^2$$

$$F_G = k_G * (x_1 - h_G)$$

2.2 Derivation of $k_{BR_{max}}$ and v_{imp} velocity

The upper limit of the range of k_{BR} was derived by equating an individual's potential energy to the elastic potential energy of a spring, given by

$$m * g * (cmi - cmh) = 0.5 * k_{BR} * (cmi - cmh)^2$$

with $cmh = 0.53 * height + h_G$ as the height of the centre of mass at the start of the fall when standing (van den Kroonenberg, Hayes, and McMahon, 1995), and $cmi = h_{HP} + h_{Femur} + h_{ST} + h_G$ as the height of the centre of mass at the start of the impact. It follows that the upper limit of the spring constant is given by $k_{BR_{max}} = \frac{-2*m*g}{cmi-cmh}$.

Calculating the impact velocity v_{imp} requires the time at impact t_{imp}, defined as the time of the hip complex, respectively, the soft tissue, starting to touch the ground. It can be obtained by solving the differential equation $m * x'' + k_{BR} * (x - cmh) + m * g = 0$ with the initial conditions $x(0) = cmh$ and $x'(0) = 0$, simulating a fall from standing height without initial velocity. By setting the solution of the differential equation to $x(t) = cmi$ and solving for t, the time at impact t_{imp} is given by

$$t_{imp} = \sqrt{\frac{m}{k_{BR}}} * \arccos\left(\frac{k_{BR} * (cmi - cmh) + g * m}{g * m}\right) \tag{1}$$

The impact velocity v_{imp} can be derived by differentiating the solution of the differential equation $x(t)$ and

solving it for $t = t_{imp}$, resulting in

$$v_{imp} = -\sqrt{\frac{m}{k_{BR}}} * g * sin\left(\sqrt{\frac{k_{BR}}{m}} * t_{imp}\right) \tag{2}$$

2.3 Strain rate correction factor

The quasi-static strain rate was taken from Guillemot *et al.* (Guillemot, 1998) and defined as $5.13 \times 10^{-4} s^{-1}$. The strain rate of a fall was calculated from analytically derived values and was set to $0.275 s^{-1}$. Using the relationship $E \sim \dot{\epsilon}^{\,0.05}$ presented by Peruzzi *et al.* (Peruzzi et al., 2021) resulted in a correction factor of 1.369. This is in line with the results of a study that compared the prediction accuracy of quasi-static finite element analysis (FEA) for the ultimate strength and stiffness values assessed experimentally in drop tower tests with thirteen proximal femora (Varga et al., 2016).

3 Soft tissue material model

We conducted two types of indentation experiments with 24 human excised soft tissue samples from twelve donors to characterise its visco-elastic behaviour. Samples were collected at the University Hospital of Graz. First, stress-relaxation experiments with different step strains (10, 25 and 40 %) and a constant loading time of 50ms were performed. And second, quasi-static force-controlled indentation experiments with a loading rate of 10 N/s up to 2000 N were conducted. The experimental data were fit with a standard non-linear solid. The forces in the non-linear springs and the damper are given by

$$F_S = slope * \log(1 + \varepsilon) + 100 * slope * \log(1 + \varepsilon)^3$$
$$F_D = slope * \frac{\varepsilon'}{(1 + \varepsilon)} + 100 * slope * \frac{\varepsilon'}{(1 + \varepsilon)} * 3 * \frac{log(1 + \varepsilon)^2}{(1 + \varepsilon)}$$

with ε describing the relative displacement as strain. The slopes were based on a spring force amplitude given by $fas = fml * \pi * r^2$ with $fml = 135$ N as the linear force module and $\pi * r^2$ with $r = 0.025m$ as the indenter area. Hence, the two spring force amplitudes are given by

$$fas_{S0} = g_{\text{inf}} * fas$$
$$fas_{S1} = g_1 * fas$$

with $g_{inf} = 0.05$ and $g_1 = 0.95$. The damping force coefficient is given by

$$\mu_{D1} = fas * g1 * \tau_1$$

with $\tau_1 = 0.025s$ being fixed. The three parameters g_{inf}, g_1, and fml were fitted using the stress-relaxation data, with $g_{\text{inf}} = 1 - g_1$. Since the quasi-static data showed a dependency of fml on the soft tissue thickness h_{ST}, a correction factor $(1/h_{ST}^2)$ was introduced.

4 CT processing and FEA

Computed tomography (CT) images were calibrated with an asynchronous calibration procedure using monthly calibration scans containing a hydroxyapatite phantom (QRM-BDC6 from QRM GmbH, Mohrendorf, Germany) with six inserts covering volumetric bone mineral density (vBMD) values ranging from 0 to 800mg/cm^3. Femur and pelvis bones were segmented using a model based on the nnU-Net method (Dudle

et al., 2023). Segmentation masks and calibrated vBMD images were used as input for a bilateral version of the vBMD pipeline described in Dudle *et al.*. The segmentation masks of both femurs are used to estimate an implicit coordinate system of the proximal femur consisting of two intersecting axes: a neck and a proximal shaft axis. The knowledge of the coordinate system is used to define a rigid body transformation of the segmentation mask in voxel space. Based on the transformed mask, a voxel mesh file is written for a load case mimicking a standardised fall to the side with the diaphysis axis inclined by 10 degrees with respect to the ground. The voxel mesh is used in a nonlinear quasi-static finite element (FE) solver with an elastic-perfectly plastic constitutive model. A load is applied to the femoral head. Femoral strength is defined as the reaction force acting on the femoral head when it reaches a vertical displacement of 4% of the vertical distance between the femoral head centre and the most lateral point of the greater trochanter in the fall configuration.

5 Soft tissue thickness detection algorithm

The detection of the soft tissue thickness is based on the same coordinate system as used for the FEA that is described in Section 4 (Dudle et al., 2023). Thereby, the greater trochanter is defined as the most inferior point of the proximal femur after transforming the mask to the standardised side fall configuration (diaphysis axis inclined by 10 degrees with respect to the ground). The soft tissue thickness was then defined as the minimum distance between the greater trochanter and the soft tissue-air boundary.

6 Substitution equations

To develop the substitution equations for the quantitative computed tomography (QCT) derived parameters, linear regression models including the variables of interest were fitted. Thereby, all available data points from the FEA analysis were included in the analysis.

6.1 Femoral height

For the substitution of femoral height h_{Femur}, the average of both sides was calculated and regressed to height. If only one side was available, this value was used.

Table 1 Results of linear regression model to substitute h_{Femur} from height

**Coefficients**	**Estimate**	**Std Error**	**t value**	**Pr**$(> \|t\|)$
(Intercept)	-0.011805	0.004875	-2.421	0.016
height	0.045121	0.002847	15.698	<2e-16

Adjusted R-squared: 0.4348. Residual standard error: 0.004518 on 318 degrees of freedom.

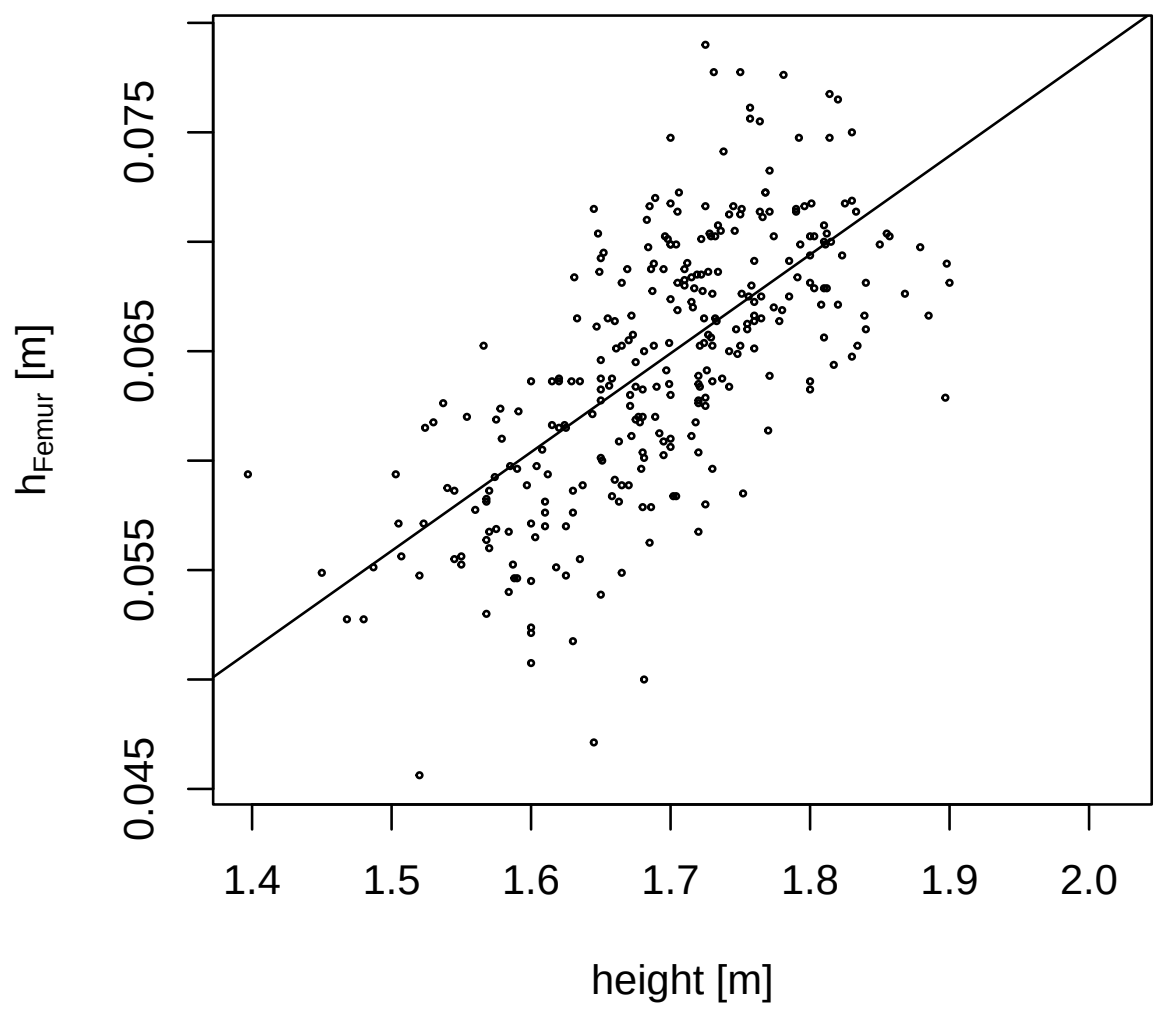

Fig. 1 Relationship between femoral height h_{Femur} and body height

6.2 Femoral strength

For the femoral strength S_{Femur}, the total hip areal bone mineral density (aBMD) was regressed to the femoral strength value of the corresponding site.

Table 2 Results of linear regression model to substitute S_{Femur} from total hip aBMD

**Coefficients**	**Estimate**	**Std Error**	**t value**	**Pr**$(>\|t\|)$
(Intercept)	8.53751	0.01389	614.83	<2e-16
log(TH aBMD)	1.56982	0.06491	24.18	<2e-16

Adjusted R-squared: 0.6554. Residual standard error: 0.1964 on 306 degrees of freedom.

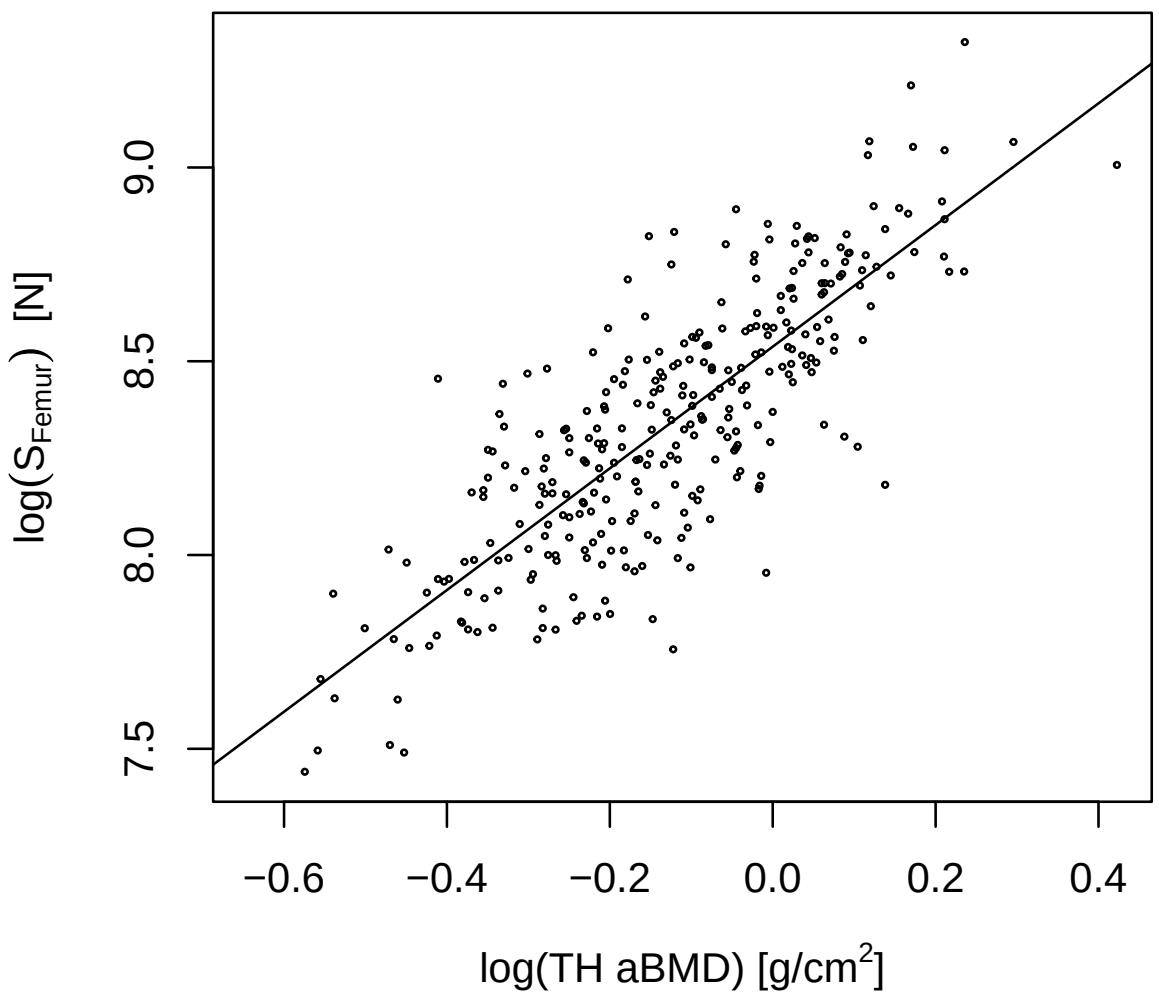

Fig. 2 Relationship between total hip aBMD and femoral strength S_{Femur}

6.3 Femoral stiffness

For the femoral stiffness k_{Femur}, the equation was derived using the FEA results of both body sides.

Table 3 Results of linear regression model to substitute k_{Femur} from S_{Femur}

**Coefficients**	**Estimate**	**Std Error**	**t value**	**Pr**$(> \lvert t \rvert)$
(Intercept)	1.464e+05	1.903e+04	7.69	5.85e-14
S_{Femur}	3.534e+02	4.083	86.56	<2e-16

Adjusted R-squared: 0.9238. Residual standard error: 154800 on 617 degrees of freedom.

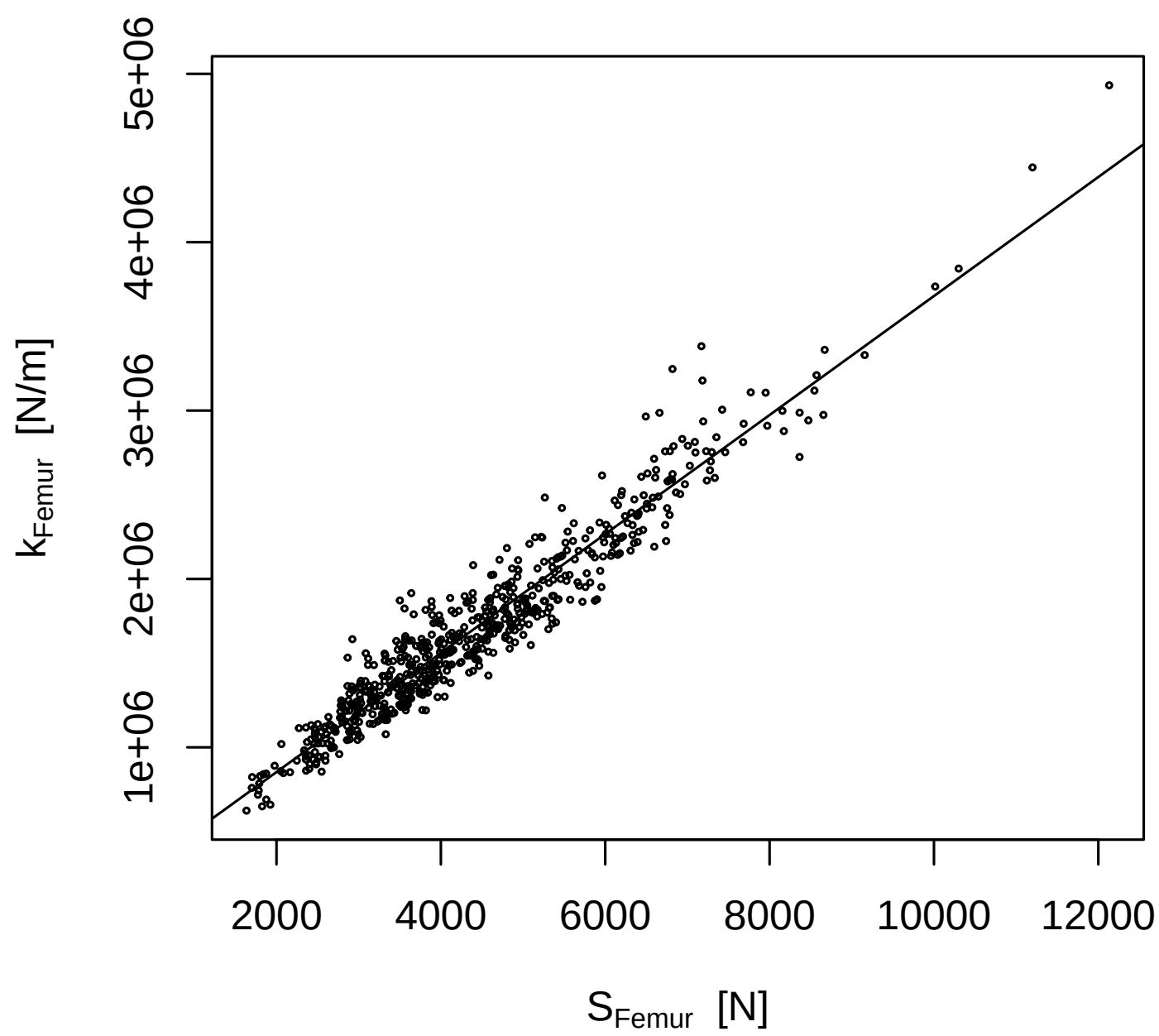

Fig. 3 Relationship between femoral strength S_{Femur} and femoral stiffness k_{Femur}

6.4 Trochanteric soft tissue thickness

For the trochanteric soft tissue thickness, the average of both sides was regressed to body mass index (BMI) and sex. If only one side was available, this value was used.

Table 4 Results of linear regression model to substitute trochanteric soft tissue thickness with BMI and sex (female as reference level)

**Coefficients**	**Estimate**	**Std Error**	**t value**	**Pr**$(>\|t\|)$
(Intercept)	-8.481	3.830	-2.214	0.0275
BMI	2.387	0.140	17.044	<2e-16
Sex:male	-19.371	1.512	-12.841	<2e-16

Adjusted R-squared: 0.5534. Residual standard error: 12.16 on 319 degrees of freedom.

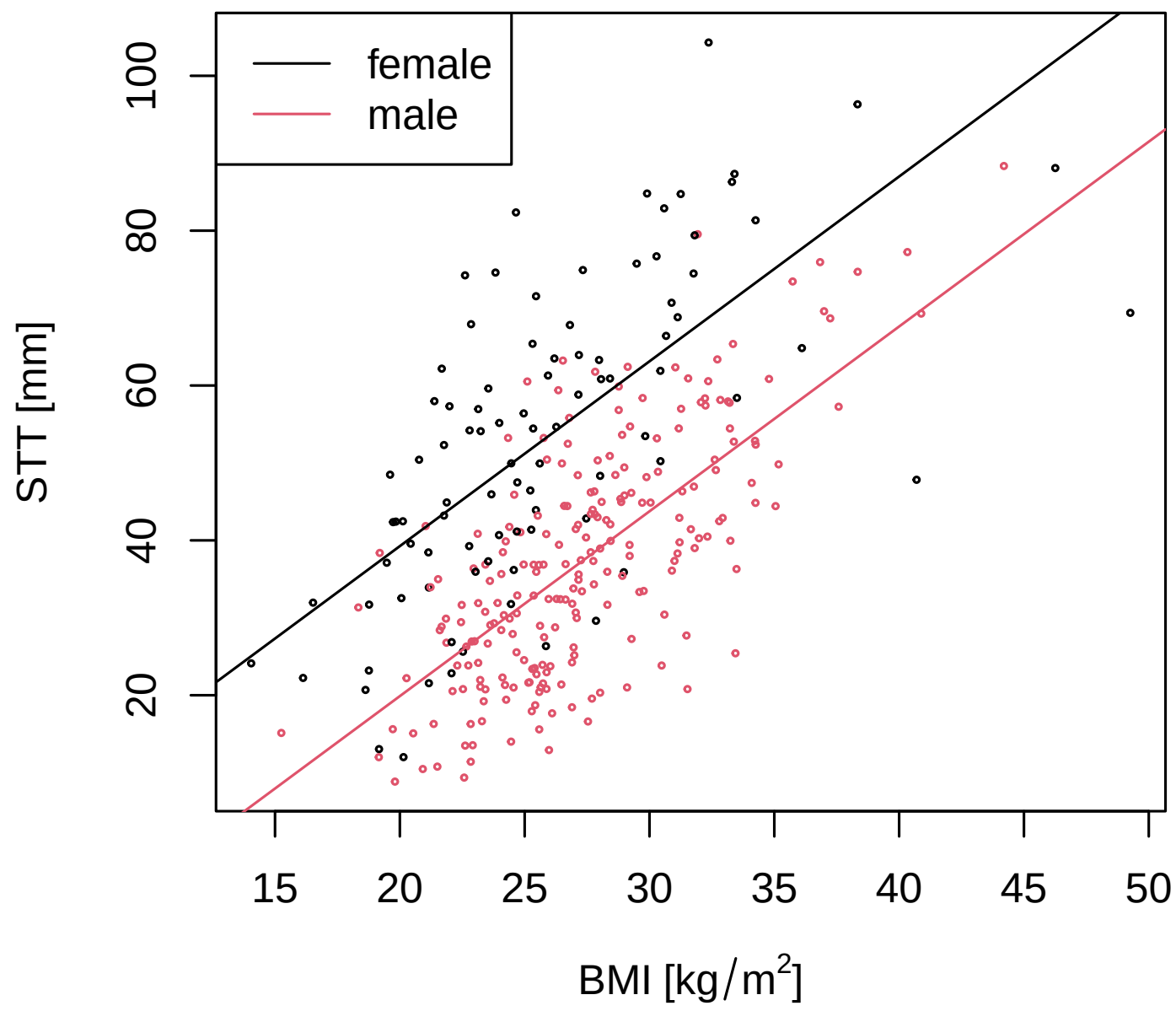

Fig. 4 Relationship between trochanteric soft tissue thickness, BMI and sex

6.5 Influence of substitution equations for femoral strength and soft tissue thickness on mechanical model parameters

The influence of the substitution equations for femoral strength and soft tissue thickness on the model output was assessed as following: The mechanical model was evaluated for a female individual with the mean values of the AFFIRM-CT cohort (weight: 78.5 kg; height: 1.69 m; h_{Femur}: 0.064 m; femoral neck aBMD: 0.89; h_{HP}), all other parameters were substituted using the equations. For the soft tissue thickness h_{ST} and the femoral strength S_{Femur}, the 95%-confidence intervals for the substituted values were calculated (lower (-1.96 standard deviations (SD) and upper (+1.96 SD) bounds for intercept and slope of the regression equations, respectively).

Table 5 Influence subsitution equation for soft tissue thickness h_{ST} on $k_{BR_{crit}}$

**SD**	h_{ST} [m]	Δh_{ST} [%]	$k_{BR_{crit}}$ [-]	Δ $k_{BR_{crit}}$ [%]
-1.96	0.042	-26 %	0.61	+14.6 %
0	0.052	-	0.53	-
1.96	0.072	+26%	0.47	-11.7 %

Table 6 Influence of femoral strength S_{Femur} subsitution equation on $k_{BR_{crit}}$

**SD**	S_{Femur} [N	ΔS_{Femur} [%]	$k_{BR_{crit}}$ [-]	Δ $k_{BR_{crit}}$ [%]
-1.96	4197	-1.23%	0.53	0 %
0	4249	-	0.53	-
1.96	4302	+1.24%	0.52	+1.46 %

While the influence of substituting the femoral strength using femoral neck aBMD with the equations from the AFFIRM-CT cohort is neglectable, estimating the soft tissue thickness using BMI introduces a bigger insecurity. Therefore, a robust method for estimating soft tissue thickness is essential.

Abbreviations

aBMD areal bone mineral density

BMI body mass index

CT computed tomography

FE finite element

FEA finite element analysis

QCT quantitative computed tomography

SD standard deviations

vBMD volumetric bone mineral density

References

Schechner, Zvi et al. (Aug. 2010). "A Poisson Process Model for Hip Fracture Risk". In: *Medical & Biological Engineering & Computing* 48.8, pp. 799–810. DOI: 10.1007/s11517-010-0638-6. (Visited on 06/11/2021).

van den Kroonenberg, A. J., W. C. Hayes, and T. A. McMahon (Aug. 1995). "Dynamic Models for Sideways Falls from Standing Height". In: *Journal of Biomechanical Engineering* 117.3, pp. 309–318. DOI: 10.1115/1.2794186.

Guillemot, H. Got (Oct. 1998). "Pelvic Behavior in Side Collisions: Static and Dynamic Tests on Isolated Pelvic Bones". In: *16th International Technical Conference on the Enhanced Safety of VehiclesNational Highway Traffic Safety AdministrationTransport CanadaTransport Canada*. Vol. 2. (Visited on 12/13/2023).

Peruzzi, Cinzia et al. (Sept. 2021). "Microscale Compressive Behavior of Hydrated Lamellar Bone at High Strain Rates". In: *Acta Biomaterialia* 131, pp. 403–414. DOI: 10.1016/j.actbio.2021.07.005.

Varga, Peter et al. (Apr. 2016). "Nonlinear Quasi-Static Finite Element Simulations Predict in Vitro Strength of Human Proximal Femora Assessed in a Dynamic Sideways Fall Setup". In: *Journal of the Mechanical Behavior of Biomedical Materials* 57, pp. 116–127. DOI: 10.1016/j.jmbbm.2015.11.026. (Visited on 06/05/2024).

Dudle, Alice et al. (Mar. 2023). "2D-3D Reconstruction of the Proximal Femur from DXA Scans: Evaluation of the 3D-Shaper Software". In: *Frontiers in Bioengineering and Biotechnology* 11, p. 1111020. DOI: 10.3389/fbioe.2023.1111020. (Visited on 06/14/2024).
